# Multi‐voxel pattern analysis of amygdala functional connectivity at rest predicts variability in posttraumatic stress severity

**DOI:** 10.1002/brb3.1707

**Published:** 2020-06-11

**Authors:** Jacklynn M. Fitzgerald, Emily L. Belleau, Tara A. Miskovich, Walker S. Pedersen, Christine L. Larson

**Affiliations:** ^1^ Department of Psychology Marquette University Milwaukee WI USA; ^2^ Department of Psychiatry McLean Hospital Belmont MA USA; ^3^ Harvard Medical School Boston MA USA; ^4^ VA Northern California Healthcare System Vallejo CA USA; ^5^ Center for Healthy Minds University of Wisconsin‐Madison Madison WI USA; ^6^ Department of Psychology University of Wisconsin‐Milwaukee Milwaukee WI USA

**Keywords:** functional magnetic resonance imaging, machine learning, multi‐voxel pattern analysis, posttraumatic stress disorder, resting state, trauma

## Abstract

**Introduction:**

Resting state functional magnetic resonance imaging (rsfMRI) studies demonstrate that individuals with posttraumatic stress disorder (PTSD) exhibit atypical functional connectivity (FC) between the amygdala, involved in the generation of emotion, and regions responsible for emotional appraisal (e.g., insula, orbitofrontal cortex [OFC]) and regulation (prefrontal cortex [PFC], anterior cingulate cortex). Consequently, atypical amygdala FC within an emotional processing and regulation network may be a defining feature of PTSD, although altered FC does not seem constrained to one brain region. Instead, altered amygdala FC involves a large, distributed brain network in those with PTSD. The present study used a machine‐learning data‐driven approach, multi‐voxel pattern analysis (MVPA), to predict PTSD severity based on whole‐brain patterns of amygdala FC.

**Methods:**

Trauma‐exposed adults (*N* = 90) completed the PTSD Checklist‐Civilian Version to assess symptoms and a 5‐min rsfMRI. Whole‐brain FC values to bilateral amygdala were extracted and used in a relevance vector regression analysis with a leave‐one‐out approach for cross‐validation with permutation testing (1,000) to obtain significance values.

**Results:**

Results demonstrated that amygdala FC predicted PCL‐C scores with statistically significant accuracy (*r* = .46, *p* = .001; mean sum of squares = 130.46, *p* = .001; *R* ^2^ = 0.21, *p* = .001). Prediction was based on whole‐brain amygdala FC, although regions that informed prediction (top 10%) included the OFC, amygdala, and dorsolateral PFC.

**Conclusion:**

Findings demonstrate the utility of MVPA based on amygdala FC to predict individual severity of PTSD symptoms and that amygdala FC within a fear acquisition and regulation network contributed to accurate prediction.

## Graphical abstract

We examined patterns of whole‐brain connectivity with the amygdala as a predictor of posttraumatic stress disorder (PTSD) severity using a machine‐learning approach. A total of *n* = 90 civilian trauma survivors completed a functional magnetic resonance imaging (fMRI) resting state scan and provided stress severity symptoms. Results showed that multi‐voxel patterns of amygdala connectivity accurately predicted severity of PTSD symptoms, while amygdala connectivity with regions involved in regulation (prefrontal cortex) contributed highly to accurate prediction.

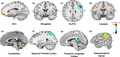

## 1 INTRODUCTION

Posttraumatic stress disorder (PTSD) is a debilitating disorder, associated with reduced physical (El‐Gabalawy, Blaney, Tsai, Sumner, & Pietrzak, 2018; Richardson, Long, Pedlar, & Elhai, 2008), occupational (Smith, Schnurr, & Rosenheck, 2005), social (Freedman, Gilad, Ankri, Roziner, & Shalev, 2015), and emotional (Radomski & Read, 2016) health and well‐being. Despite the negative impact of PTSD on overall quality of life (Vogt et al., 2017), the neurophysiology of this disorder is still not well understood. Prior research implicates atypical amygdala response and amygdala functional connectivity (FC) as a cardinal feature of the disorder (Liberzon & Sripada, 2007; Patel, Spreng, Shin, & Girard, 2012; Rauch, Shin, & Phelps, 2006). In regard to FC studies, findings suggest that the amygdala is atypically connected with a number of brain areas spanning both cortical and subcortical regions (Bryant et al., 2008; Diener et al., 2016; Felmingham et al., 2014; Fonzo et al., 2010; Hayes et al., 2011; Hendler et al., 2003; Killgore et al., 2014; Linnman, Zeffiro, Pitman, & Milad, 2011; Nilsen et al., 2016; Patel, Girard, Pukay‐Martin, & Monson, 2016; Shin et al., 2005; Simmons et al., 2011; St. Jacques, Botzung, Miles, & Rubin, 2011; Williams et al., 2006). This suggests that amygdala FC changes in PTSD are widespread. As such, whole‐brain amygdala FC may be a meaningful biomarker of PTSD severity, although this has yet to be tested. As interest in precision medicine grows (Collins & Varmus, 2015), more research is needed on the amygdala and its broader connectivity across the brain in those with PTSD in order to assess whether this may be meaningfully related to the disorder and provide insight into treatment can lead to remediation of symptoms.

The amygdala is active in response to motivationally relevant stimuli, specifically stimuli that convey threat or danger (Costafreda, Brammer, David, & Fu, 2008). Early neuroimaging work from both functional magnetic resonance imaging (fMRI) and positron emission tomography (PET) studies report that the amygdala is hyper‐responsive to negative faces (Bryant et al., 2008; Felmingham et al., 2010; Fonzo et al., 2010; Killgore et al., 2014; Rauch et al., 2000; Shin et al., 2005; Simmons et al., 2011), scenes (Brohawn, Offringa, Pfaff, Hughes, & Shin, 2010; Brunetti et al., 2010; Patel et al., 2016), words (St. Jacques et al., 2011), and trauma‐specific stimuli (Hendler et al., 2003; Peres et al., 2011; Protopopescu et al., 2005) in those with PTSD compared to both trauma‐exposed and healthy controls. Aberrant amygdala FC in response to threat also occurs in those with PTSD and spans a broad neural network. For instance, in response to threat, individuals with PTSD exhibit aberrant amygdala–brainstem (Steuwe et al., 2015), amygdala–thalamus (Morey et al., 2015; Rabellino et al., 2016), amygdala–medial prefrontal cortex (MPFC) and amygdala–anterior cingulate cortex (ACC) connectivity (Cisler, Scott Steele, Smitherman, Lenow, & Kilts, 2013; Keding & Herringa, 2016; Neumeister et al., 2016; Rabellino et al., 2016; Stevens et al., 2013; White, Costanzo, Blair, & Roy, 2015; Wolf & Herringa, 2016). Both healthy controls and traumatized controls are used as comparison groups throughout the literature, with no clear relationship between the directionality of findings and the type of control group employed. Individual differences in altered amygdala FC (e.g., either decreased or increased) also correspond to PTSD symptom severity (Cisler et al., 2013; Keding & Herringa, 2016; Stevens et al., 2013; White et al., 2015; Wolf & Herringa, 2016) and in instances where authors did not find a relationship between PTSD symptom severity and amygdala *activation* (Keding & Herringa, 2016). Further, severity of PTSD symptoms correlates with amygdala FC in trauma survivors without a PTSD diagnosis (Cisler et al., 2013; Stevens et al., 2013) and in individuals with sub‐threshold severity of symptoms (White et al., 2015). Thus, amygdala FC appears to be a sensitive biomarker for individual differences in PTSD symptom severity.

During rest (e.g., when not viewing threatening stimuli), individuals with PTSD also display aberrant amygdala FC (either increased or decreased compared to controls) with the insula (Nicholson et al., 2016; Rabinak et al., 2011; Sripada et al., 2012; X. Zhang, Wu, et al., 2016) orbitofrontal cortex (OFC [(Aghajani et al., 2016; Zhang, Wu, et al., 2016; Zhu et al., 2017), MPFC (Aghajani et al., 2016; Brown et al., 2014; Jin et al., 2014), and hippocampus (Li et al., 2017; Sripada et al., 2012). Direction of findings does not appear to depend on within structure differentiations (e.g., comparing dorsal vs. ventral MPFC) (Aghajani et al., 2016). Differences in the direction of amygdala FC in those with PTSD (e.g., increased, decreased) may be caused by differences in amygdala subnuclei, as the basolateral amygdala (BLA) and centromedial amygdala (CMA) have different functions (Phelps, 2004). Yet, to date, only a handful of unique studies have investigated FC with amygdala subnuclei (*N = 7*) and again report disparate findings, specifically differential FC patterns for BLA compared to CMA (Brown et al., 2014; Koch et al., 2016; Nicholson et al., 2015, 2017; Varkevisser, Gladwin, Heesink, van Honk, & Geuze, 2017; Zhu et al., 2017) or no differences between the subnuclei (Zhu et al., 2018), while no study reports identical aberrations in amygdala FC by subregion. Alongside failure to replicate, across all studies, altered FC is diffuse and aberrations span the frontal cortex, cingulate, parietal lobe, thalamus, cerebellum, and precuneus. Heterogeneous findings in terms of direction (e.g., increased, decreased) and brain location demonstrate a need for better precision in mapping atypical neural networks in PTSD.

Nevertheless, PTSD symptom severity correlates with atypical amygdala FC (Aghajani et al., 2016; Jin et al., 2014; Keding & Herringa, 2016; Li et al., 2017; Nicholson et al., 2016; Zhang, Wu, et al., 2016; Zhu et al., 2017). Other work shows that amygdala FC changes as a function of treatment response and thus remains a good target for the pathophysiology of the disorder. Decline in PTSD symptoms after trauma‐focused cognitive behavioral therapy (TF‐CBT) (Cisler et al., 2016), CBT (Shou et al., 2017), and prolonged exposure (PE) (Stojek, McSweeney, & Rauch, 2018; Zhu et al., 2018) all correlate with amygdala FC. Pretreatment amygdala FC also predicts clinical improvement after transcranial magnetic stimulation (Philip et al., 2018). Although not in the context of FC, other research shows that amygdala activation in response to threat correlates with PTSD severity even in cases where amygdala activation did not differ by group (PTSD vs. Control) (El Khoury‐Malhame et al., 2011). Amygdala activation and its connectivity are therefore a reliable measure of state‐dependent symptom severity, while changes in amygdala FC as a function of treatment occur across treatment modalities. However, despite several studies investigating amygdala FC as a “neurobiological” marker of PTSD disease state, prior work has overwhelmingly used a univariate approach to examine the relationship between rsfMRI amygdala FC with a single region (e.g., insula or PFC) and symptoms. As the above findings demonstrate, those with PTSD exhibit altered amygdala FC with a large brain network spanning limbic and cortical regions. Therefore, an alternative approach is to investigate whether patterns of *distributed* amygdala connectivity accounts for variability in stress symptomatology.

Multi‐pattern voxel analyses (MVPA) have gained traction in recent years as a way to map spatially distributed patterns of brain activation and/or FC (Cohen et al., 2017; Pereira, Mitchell, & Botvinick, 2009). Rather than testing the association between symptoms and discrete reactivity or FC (i.e., within or between a couple of regions), this approach examines whether whole‐brain distributed neural activation patterns are correlated with symptoms, leveraging the fact that brain functioning is defined by spatially distributed processes (Davis et al., 2014). In adding machine learning as an analysis technique, MVPA can be used to subsequently predict individual differences in symptom severity based on a spatially extensive pattern of activation in the brain (Clark et al., 2014). To date, relatively few studies have used MVPA and machine learning to study the association between neural functioning and individual differences in PTSD symptom severity, although this approach has been used successfully in patients with depression (Habes et al., 2013; Mwangi, Matthews, & Steele, 2012; Yang et al., 2016) and anxiety (Visser, Haver, Zwitser, Scholte, & Kindt, 2016). In the context of PTSD, Liu and colleagues used MVPA and a machine‐learning method known as support vector machine (SVM) to demonstrate that whole‐brain amplitude of low‐frequency fluctuations (ALFF) and whole‐brain FC based on 116 regions of interest predicts patients from controls with 93% accuracy (Liu et al., 2015). Zhang and colleagues found similar results, using whole‐brain ALFF to predict patients from controls with 89% accuracy (Zhang, Zhang, Zhang, Wang, Li, & Zhang, 2016). Gong and colleagues used MVPA and SVM to also demonstrate that whole‐brain patterns of structural integrity accurately predict patients with PTSD from healthy controls with 91% accuracy (Gong, Li, Du, et al., 2014). In a follow‐up paper, this group of researchers also found that whole‐brain rsfMRI ALFF predicted individual differences in PTSD severity using another machine‐learning technique called relevance vector regression (RVR) (Gong, Li, Tognin, et al., 2014). As opposed to SVM, RVR utilizes a regression approach to test whether distributed neural patterns can accurately predict individual differences in symptom severity, rather than predicting a dichotomous classification such as PTSD diagnosis. Altogether, these publications demonstrate that MPVA and machine‐learning approaches can be used to accurately distinguish those with PTSD and predict individual differences in PTSD symptom severity. However, these studies did not examine whether whole‐brain amygdala FC at rest also predicts PTSD symptom severity.

The current study used MVPA and machine learning to predict PTSD severity based on whole‐brain patterns of amygdala FC collected from rsfMRI. Previous studies have found that PTSD severity is related to amygdala FC with a large host of brain regions. That is, based on pre‐existing literature, amygdala FC with any number of subcortical and cortical regions is associated with severity of symptoms. By focusing on singular brain regions or even singular neurocircuitries (e.g., frontoparietal network), this research may ignore larger‐scale dysfunction in amygdala FC across the entire brain as an indication of PTSD severity. Based on prior literature, we hypothesized that whole‐brain patterns of amygdala FC would accurately predict individual differences in PTSD severity in a sample of trauma‐exposed adults. Based on evidence of aberrant amygdala FC to regions involved in fear learning and regulation, we further hypothesized that while results would be informed by whole‐brain (e.g., global) amygdala FC, amygdala FC to regions instrumental for mounting a fear response (i.e., brainstem, thalamus, insula, hippocampus, OFC) and regulation of this response (i.e., MPFC, ACC) would be among the top regions that contributed to PTSD severity.

## 2 MATERIAL AND METHODS

### 2.1 Participants

Ninety‐two undergraduate Caucasian adults were recruited at the University of Wisconsin‐Milwaukee (Milwaukee, WI). Participants were deemed eligible if they were between the ages of 18–50, had normal or corrected‐to‐normal vision, were right‐handed, a Native English speaker, able to provide informed consent, and endorsed personally experiencing a trauma as reported on the Life Events Checklist (LEC) (Gray, Litz, Hsu, & Lombardo, 2004). All participants completed the Mini‐International Neuropsychiatric Interview (M.I.N.I. [Sheehan et al., 1997]), and participants were excluded if they had a clinically significant neurological disorder, history of seizures or head injuries, endorsed symptoms of mania, schizophrenia, obsessive‐compulsive disorder, or panic attacks. Participants were also excluded if they were currently taking antipsychotics, anticonvulsants, or mood stabilizers. Due to the use of MRI scanning, participants were excluded if they were deemed MRI incompatible based on the presence of ferromagnetic material in the body, claustrophobia, were unable to lie still for two hours, or were pregnant or trying to become pregnant. Participant demographics are listed in Table 1. All participants completed a consent form approved by the local Institutional Review Board at the University of Wisconsin‐Milwaukee. Participants were compensated for their time and all procedures complied with the Helsinki Declaration.

**Table 1:** Sample demographics (*N* = 90)

	*M *(*SD*)
Age	22.12 (3.72)
PCL‐C	31.10 (12.93)

	*n *(%)
Gender (Female)	62 (68.90%)
Diagnoses	
Agoraphobia	4 (4.44)
Alcohol use disorder (AUD)	13 (14.44%)
Attention‐deficit hyperactivity disorder (ADHD)	1 (1.11%)
Generalized anxiety disorder (GAD)	7 (7.78%
Major depressive disorder (MDD)	5 (5.56%)
Posttraumatic stress disorder (PTSD)	2 (2.22%
Social anxiety disorder (SAD)	1 (1.11%)
Substance use disorder (SUD)	5 (5.56%)
Trauma Exposure as reported on the Life Events Checklist (LEC)	
Natural disaster	15 (16.70%)
Fire or explosion	13 (14.40%)
Transportation accident	64 (71.10%)
Serious accident at work, home, or during recreational activity	18 (20.00%)
Exposure to toxic substance	8 (8.90%)
Physical assault	36 (40.00%)
Assault with a weapon	8 (8.90%)
Sexual assault	15 (16.70%)
Other unwanted or uncomfortable sexual experience	29 (32.20%)
Combat or exposure to a war zone	1 (1.10%)
Captivity	2 (2.20%)
Life‐threatening illness or injury	5 (5.60%)
Severe human suffering	3 (3.30%)
Sudden, violent death	2 (2.20%)
Sudden, unexpected death of someone close to you	40 (44.40%)
Serious injury, harm, or death you caused to someone else	3 (3.30%)
Any other very stressful event or experience	39 (43.30%)

Note

Diagnoses and trauma exposures are not mutually exclusive.

Abbreviation: PCL‐C, PTSD Checklist‐Civilian Version.

### 2.2 Measure of PTSD symptom severity

Symptoms of PTSD were acquired using the PTSD Checklist‐Civilian Version based on the fourth edition of the Diagnostic and Statistical Manual of Mental Disorders (Weathers, Litz, Huska, & Keane, 1994). The PCL‐C is a 17‐item self‐report measure of stress symptoms with good internal consistency (Cronbach's *α* = 0.94), convergent validity (*r* > .75), and test–retest reliability (*r* = .92) (Ruggiero, Ben, Scotti, & Rabalais, 2003), including in nonclinical samples to assess stress severity (Conybeare, Behar, Solomon, Newman, & Borkovec, 2012). Use of the PCL‐C to quantify PTSD severity is consistent with prior publications using MVPA and machine learning in this population (Gong, Li, Du, et al., 2014; Gong, Li, Tognin, et al., 2014).

### 2.3 Resting state fMRI acquisition

All participants completed a 5‐min resting state scan during fMRI. During the scan, participants viewed a white crosshair displayed on a black background and were instructed to keep their eyes open. Scanning was performed on a 3.0 Tesla short bore GE Signa Excite MRI system at the Medical College of Wisconsin. Functional T2*‐weighted echoplanar images (EPI) were collected in a sagittal orientation with the following parameters: repetition time (TR)/echo time (TE) = 2,000/25 ms; FOV = 24 mm; matrix = 64 × 64; flip angle = 77°; slice thickness = 3.5 mm. A high‐resolution T1‐weighted anatomical image was also acquired for co‐registration with the following parameters: TR/TE = 8.2/3.2 ms; FOV = 240 mm; matrix = 256 × 224; flip angle = 12°; voxel size = 0.9375 × 1.071 × 1 mm.

### 2.4 Data analysis

#### 2.4.1 Image preprocessing

Individual functional images were analyzed using the CONN FC toolbox (Whitfield‐Gabrieli & Nieto‐Castanon, 2012). Images were preprocessed according to standard procedures. Briefly, images underwent spatial realignment using the SPM12 *realign and unwarp* procedure (Andersson, Hutton, Ashburner, Turner, & Friston, 2001) with all scans referenced to the first image and estimated motion parameters calculated across six variables representing, to be used as regressors of no interest. Temporal misalignment was corrected using slice time correction (Henson, Buchel, Josephs, & Friston, 1998). As small head movements can cause spurious noise‐ and distance‐dependent changes in signal correlations (Power et al., 2014; Power, Schlaggar, & Petersen, 2015), frame‐wise displacement (FD) was computed to rule out confounding effects of motion. Volumes with FD > 0.2 mm (plus 1‐back and 2‐forward neighboring volumes) were “scrubbed” (e.g., removed from analysis), and subjects with >3 mm or 3° of rotational cumulative movement were dropped from analysis. Structural segmentation and normalization were done to classify data into gray matter, white matter, cerebrospinal fluid (CSF) through the estimation of the posterior tissue probability maps in SPM12 (Ashburner & Friston, 2005). Images were then normalized to the Montreal Neurological Institute template and smoothed with a 4 mm^3^ Gaussian kernel (Hagler, Saygin, & Sereno, 2006). To isolate rsfMRI signal, resulting data were bandpass filtered at 0.01–0.09 Hz, while signal from CSF, white matter, and motion realignment parameters were entered as regressors of no interest to control for these effects during scanning.

#### 2.4.2 Pattern recognition analysis

Using CONN, whole‐brain bilateral amygdala FC maps were computed at the first level (e.g., within‐subjects) for each individual using the anatomical automatic labeling (AAL)‐defined bilateral amygdala mask from the SPM toolbox (Maldjian, Laurienti, Kraft, & Burdette, 2003; Tzourio‐Mazoyer et al., 2002) as the seed region. This produced an amygdala FC map for each individual, where each voxel represented a Fisher‐transformed bivariate correlation coefficient between bilateral amygdala BOLD time series and every other voxel's BOLD time series. In traditional mass‐univariate statistical approaches, these maps are subsequently used in second‐level (e.g., between‐subjects) analyses of connectivity values to investigate the relationship between spatially discrete amygdala FC values (e.g., within certain brain regions) and PCL‐C scores. Instead, we used each individual amygdala FC map and a multivariate RVR approach using the PRoNTo toolbox ([Schrouff, Rosa, et al., 2013]; http://www.mlnl.cs.ucl.ac.uk/pronto/) to statistically test whether the whole‐brain pattern of amygdala FC (e.g., across all voxels) predicted PCL‐C scores.

In contrast to SVM methods that predict classification of groups based on MVPA, RVR is a pattern recognition method that uses Bayesian inference to obtain sparse regression models (Tipping, 2001). Sparsity is achieved in the classification of zero versus nonzero weights through the calculation of the Bayesian posterior distribution of all weights. In this process, the majority of weights peak at zero with relatively few nonzero weights, which are subsequently used to define parameter optimization. To constrain the maximum likelihood estimation of this model in this way, the weight distribution is applied with a zero‐mean Gaussian prior probability distribution (Tipping, 2001). The posterior distribution that is optimized in this process is then used to predict target values (e.g., PCL‐C score) from amygdala FC maps. In effect, this method is used to predict continuous characteristics from patterns of neuroimaging data weighted for relevance (Hou et al., 2016; Stonnington et al., 2010). In the RVR approach, training (“relevance”) vectors establishing all model weights are iteratively estimated, and only the model weights (e.g., nonzero) that are deemed relevant based on training data remain in the model (Formisano, De Martino, & Valente, 2008). Unlike SVM approaches, RVR is a sparse kernel method, and therefore, the number of relevance vectors used for model estimation does not automatically linearly grow with size of the training set.

For the current analysis, one image representing bilateral amygdala FC maps for each individual was used for feature selection, with amygdala FC representing connectivity across the entire scan duration. Feature selection was constrained to voxels inside the brain through the use of a standard binary mask (Schrouff, Rosa, et al., 2013). In the calculation of features, a linear kernel was used with a square matrix of dimensions N × N, where the kernel reflected a similarity measure between each participant, called the dot product. No second‐level mask was used to constrain feature selection by a subset of voxels; instead, all voxels were used to compute features. In model specification, we used the RVR approach, described above. In this process, features were mean‐centered using the training data and generalizability of the model was estimated using a leave‐one‐out approach for cross‐validation. Cross‐validation is used to ensure generalizability of the model and to not overfit the data. The performance of the model was characterized using the (cross‐validated) Pearson correlation coefficient (*r*), mean squared error (*MSE*), and the coefficient of determination (*R* ^2^) between estimated and true PCL‐C scores. Significance values for prediction scores were obtained using permutation testing (1,000 iterations), a necessary step when dealing with large neuroimaging datasets that violate the assumption that data are independently and identically distributed. The choice for 1,000 permutations is identical with the methods found in with prior machine‐learning MVPA publications using neuroimaging data (Gong, Li, Du, et al., 2014).

To view results of the model, colormaps were created that reflected the contribution of each voxel, representing bilateral amygdala FC values, toward model performance. Voxels with high weight values, represented by warmer colors, indicate that these regions positively contributed to model performance. In contrast, voxels with low weight values, represented by cooler colors, indicate weight values that negatively contributed to model performance (e.g., push it toward decreased prediction). Post hoc averaging of weight values by individual brain regions was also done during visualization of results (Schrouff, Cremers, et al., 2013). For averaging by brain region, we utilized the AAL atlas, resulting in the averaging of weight values within *N* = 117 brain regions.

## 3 RESULTS

### 3.1 Participants

Two participants were excluded due to excessive motion (>3 mm in any direction) during rsfMRI, leaving a total of 90 participants available for data analysis.

### 3.2 Trauma exposure

All participants endorsed personally experiencing at least one traumatic event based on LEC scores as stipulated in the inclusion criteria. However, LEC scores indicated that 85.60% of participants endorsed personally experiencing multiple traumas, while 14.40% endorsed experiencing a single traumatic event. The three most frequent types of traumas reported were as follows: transportation accidents (71.10%), sudden and unexpected death of someone close (44.40%), and physical assault (40.00%). Table 1 includes a detailed listing of LEC trauma types and frequencies. To note, trauma types are not mutually exclusive across participants, reflecting high incidence of multiple traumas in this sample.

### 3.3 PTSD symptoms

Posttraumatic stress disorder symptoms as measured by PCL‐C scores ranged from 17 to 75 (*M* = 31.10, *SD* = 12.93) indicating variability in PTSD symptom severity from minimal to moderate/severe and a good distribution in scores. Using a recommended > 30 PCL‐C cut‐point score (Blanchard, Jones‐Alexander, Buckley, & Forneris, 1996), 45.60% of the sample were eligible for a PTSD diagnosis.

### 3.4 MVPA results

**Figure 1:**
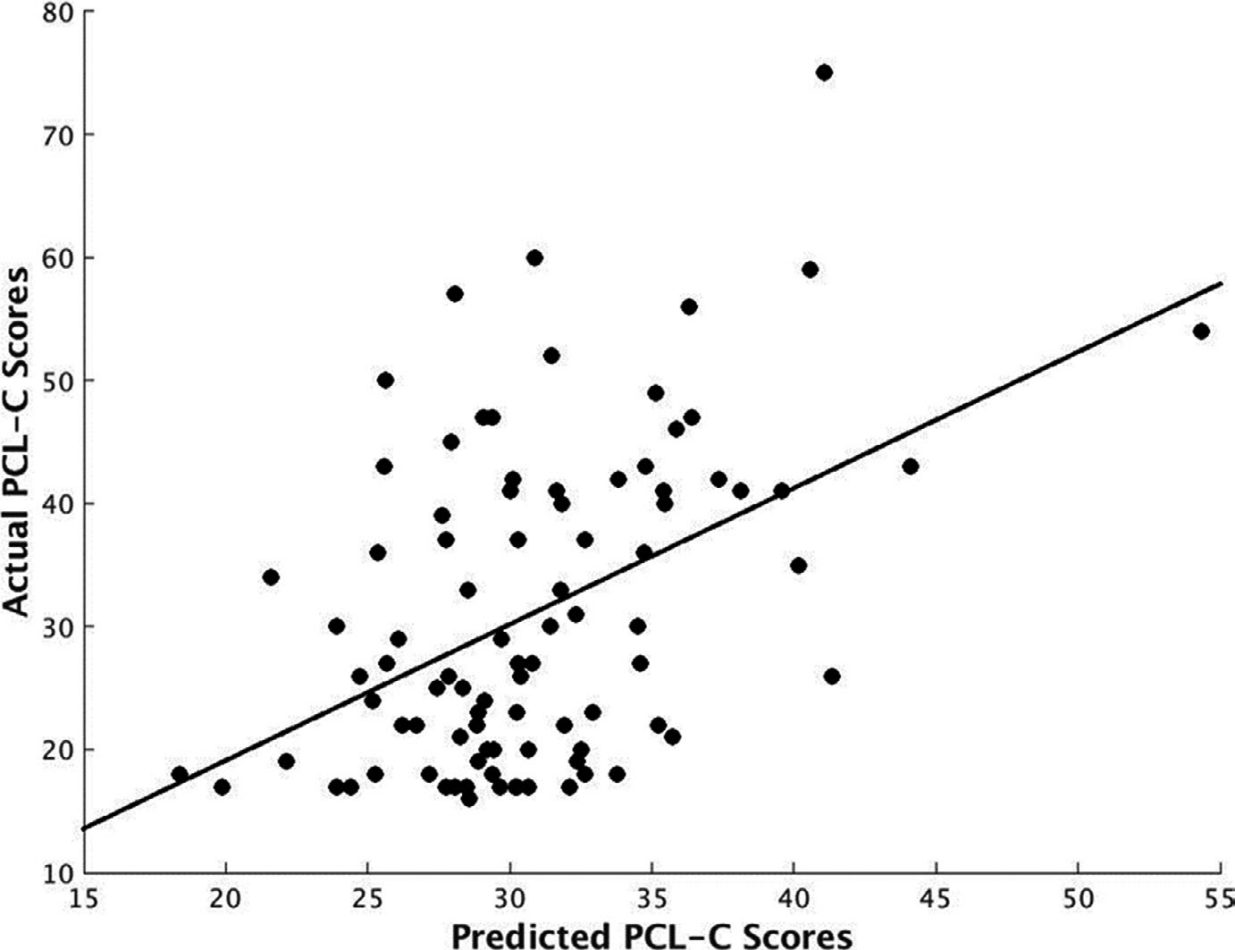
Significant relationship between actual and predicted PCL‐C scores based on the MVPA algorithm (*r* = .46, *p* = .001; mean sum of squares = 130.46, *p* = .001; *R* ^2^ = 0.21, *p* = .001). MVPA, multi‐pattern voxel analysis; PCL‐C, Posttraumatic stress disorder Checklist‐Civilian Version

Amygdala FC predicted PCL‐C scores with statistically significant accuracy (*r* = 0.46, *p* = .001; mean sum of squares = 130.46, *p* = .001; *R* ^2^ = 0.21, *p* = .001), while prediction was based on amygdala FC across the whole brain. As our sample was unequal in gender distribution (68.90% female), we re‐ran analyses controlling for gender. Results were unchanged with almost no deviation in the strength of this relationship, such that amygdala FC remained a significant predictor of PCL‐C scores (*r* = .48, *p* = .001; mean sum of squares = 128.43, *p* = .001; *R* ^2^ = 0.23, *p* = .001). Given high concordance between PTSD and MDD and the need for specificity in isolating prediction for PTSD severity (Flory & Yehuda, 2015), we also re‐ran analyses controlling for diagnosis of MDD; results remained significant (*r* = .51, *p* = .001; mean sum of squares = 123.30, *p* = .001; *R* ^2^ = 0.26, *p* = .002). Figure 1 depicts the relationship between actual PCL‐C scores on the y‐axis plotted against predicted PCL‐C scores based on the MVPA algorithm on the *x*‐axis. In plotting this relationship, we identified two possible outliers based on actual or predicted PCL‐C scores. We subsequently removed these individuals and re‐ran analyses on the *N* = 88 remaining participants. Results remained unchanged and amygdala FC was still a significant predictor of PTSD severity (*r* = .30, *p* = .006; mean sum of squares = 128.28, *p* = .006; *R* ^2^ = 0.09, *p* = .047).

**Figure 2:**
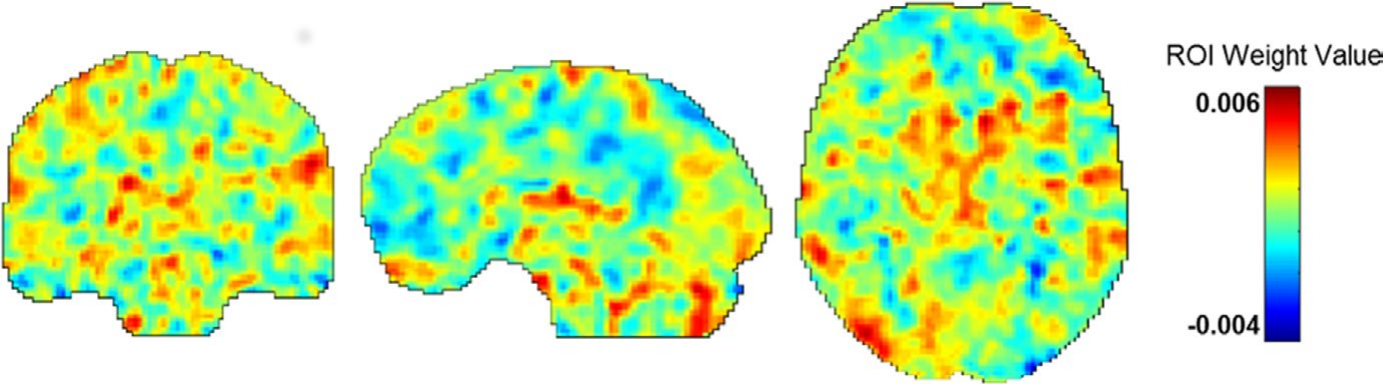
Results of the RVR analysis depicting weight value for each voxel. RVR, relevance vector regression

**Figure 3:**
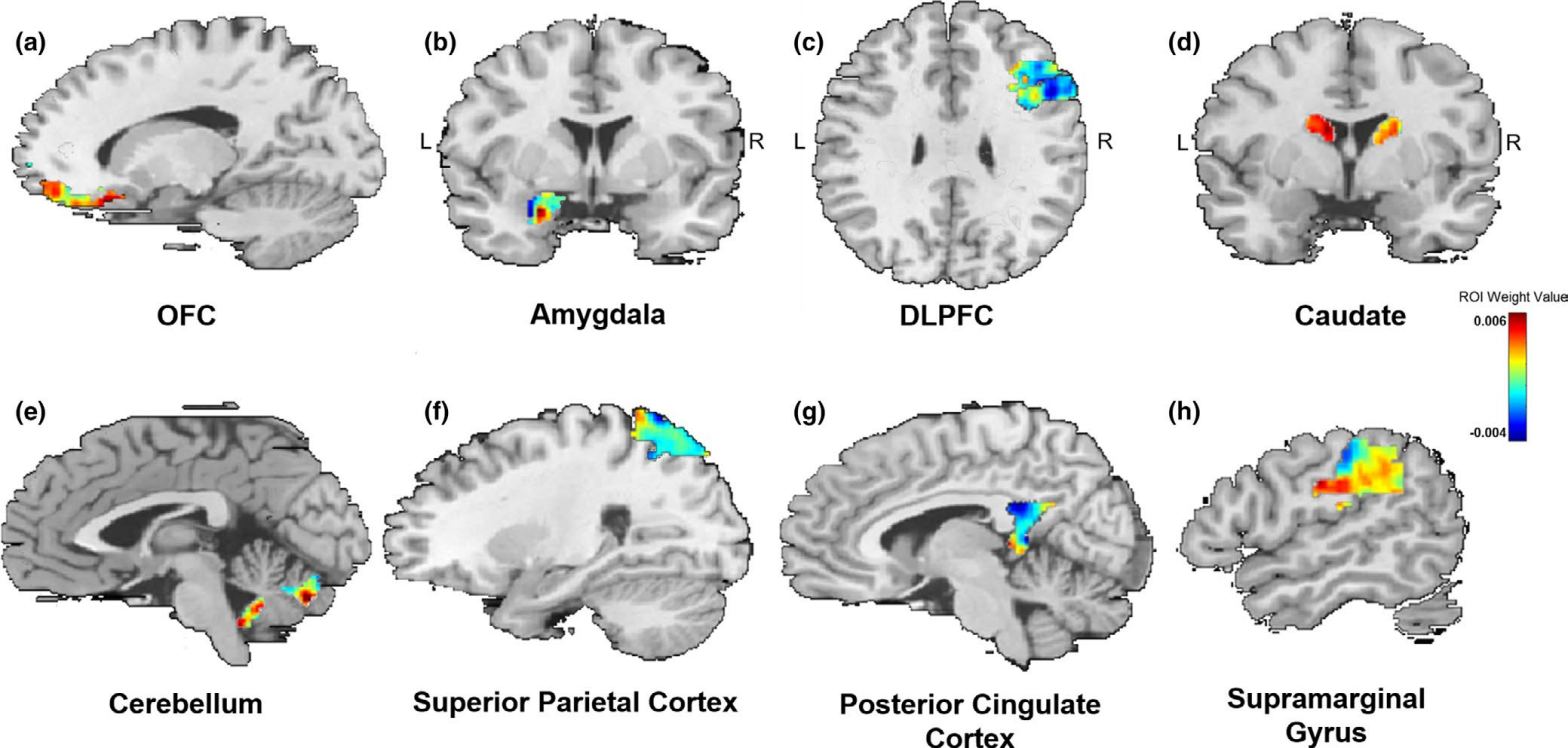
Spatial location of top 10% of weighted regions that predicted PCL‐C scores. The (a) OFC and (b) amygdala are involved in the acquisition of fear. Conversely, the (c) DLPFC is involved in the regulation of fear. Additionally, the (d) caudate, (e) cerebellum, (f) superior parietal cortex, (G) posterior cingulate cortex, and (h) supramarginal gyrus were among the top regions that contributed to the model. DLPFC, dorsolateral prefrontal cortex; OFC, orbitofrontal cortex; PCL‐C, Posttraumatic stress disorder Checklist‐Civilian Version; R, right; L, left

Similar to others (Gong, Li, Du, et al., 2014; Hou et al., 2016), we used a 10% threshold to visualize RVR‐derived weights, which resulted in a listing of regions with the greatest weight vector values. Regions implicated in fear acquisition and regulation, including the OFC, amygdala, and the dorsolateral prefrontal cortex (DLFPC), were within this top 10% (Table 2). Figure 2 displays results of the RVR analysis depicting weight value for each voxel representing bilateral amygdala FC, while Figure 3 provides spatial location of regions within the top 10% (e.g., with greatest weight vector values). A distribution of relevant model weights by regions in the atlas was also produced (Figure S1), which provided more information on spatial location of relevant model weights. The x‐axis of the distribution demonstrated that averaged model weights contributing to model estimation spanned all brain regions.

**Table 2:** Model weights per regions of interest

Region of Interest	Laterality	Weight (%)	Size (voxels)	Expected Ranking	MNI Coordinates		
					*x*	*y*	*z*
Cerebellar vermis	Midline	1.56	105	3.13	0	−46	−32
Caudate	L	1.55	942	2.11	−12	12	10
Caudate	R	1.45	982	3.14	14	14	10
**DLPFC**	**R**	**1.40**	**1,208**	**4.67**	**48**	**14**	**22**
Superior parietal cortex	R	1.30	1,471	6.14	**24**	**−58**	**60**
Cerebellar vermis	Midline	1.29	195	6.52	2	−72	−26
**OFC**	**R**	**1.25**	**556**	**7.00**	**18**	**46**	**−14**
**DLPFC**	**R**	**1.21**	**1,559**	**9.61**	**46**	**28**	**14**
PCC	R	1.14	323	11.78	6	−42	24
**Amygdala**	**L**	**1.13**	**211**	**9.64**	**−24**	**0**	**−16**
Supramarginal gyrus	R	1.12	1,598	16.28	56	−32	34

Note

Reported regions represent top 10% of regions based on weight. Weight is determined by the contribution of that region divided by the total contribution of all regions and displayed as a percentage. Expected ranking reflects how stable the ranking of each region is across folds. Bolded text reflects regions of interest involved in acquisition and regulation of fear.

Abbreviations: DLPFC, dorsolateral prefrontal cortex; L, left; MNI, Montreal Neurological Institute; OFC, orbitofrontal cortex; PCC, posterior cingulate cortex; R, right.

## 4 DISCUSSION

The current study used an MVPA approach to determine whether whole‐brain patterns of bilateral amygdala FC predicted individual differences in PTSD severity in an adult trauma‐exposed sample. Several insights emerged from this investigation: First, whole‐brain patterns of amygdala FC did significantly predict severity of PTSD symptoms, indicating that whole‐brain patterns of amygdala connectivity are meaningfully related to variability in PTSD outcomes in trauma‐exposed individuals. Second, connectivity to regions involved in fear acquisition (i.e., amygdala), appraisal (i.e., OFC), and regulation (e.g., DLPFC) were among the top regions most helpful for predicting PTSD severity. The evidence from this data‐driven approach supports existing theoretical frameworks outlining the importance of regions implicated in fear dysregulation for the etiology of PTSD (Liberzon & Sripada, 2007; Patel et al., 2012; Rauch et al., 2006).

Principally, these findings demonstrate that amygdala FC across the entire brain, versus with discrete regions, is helpful at predicting variability in PTSD severity in a sample of trauma‐exposed adults. In focusing on a distributed pattern of activation in this analysis, we have demonstrated that connectivity patterns across the entire brain may be a more precise biomarker for severity of PTSD symptoms, at least in some cases. Based on these findings, prior studies that did not find a relationship between symptom severity and brain response (Bryant et al., 2008; Diener et al., 2016; Felmingham et al., 2014; Fonzo et al., 2010; Hayes et al., 2011; Hendler et al., 2003; Killgore et al., 2014; Linnman et al., 2011; Nilsen et al., 2016; Patel et al., 2016; Shin et al., 2005; Simmons et al., 2011; St. Jacques et al., 2011; Williams et al., 2006) or between symptom severity and amygdala FC (Rabinak et al., 2011; Sripada et al., 2012) may benefit from investigating the relationship between symptoms and distributed patterns of activation. By demonstrating sensitivity of whole‐brain amygdala FC to predict individual variation in PTSD symptoms, the current study replicates prior research that also found whole‐brain measures of functional activity (e.g., rsfMRI ALFF) (Gong, Li, Tognin, et al., 2014; Liu et al., 2015; Zhang, Zhang, et al., 2016) and whole‐brain gray matter volume (Gong, Li, Du, et al., 2014; Zhang, Zhang, et al., 2016) useful for predicting PTSD illness. Altogether, this demonstrates that whole‐brain data‐driven approaches have merit for mapping the neurobiological underpinnings associated with PTSD.

In addition, we also found evidence that regions involved in fear acquisition (i.e., amygdala), appraisal (i.e., OFC), and regulation (i.e., DLPFC) were among the top regions that predicted PTSD severity. As stated prior, these regions are studied extensively in the context of PTSD (Liberzon & Sripada, 2007; Patel et al., 2012; Rauch et al., 2006) and our results further confirm their clinical significance for the disorder. Based on these results, intra‐amygdala connectivity may be an important predictor of PTSD status. This is supported by recent work that has found fine‐grained structural abnormalities within the amygdala in those with PTSD (Akiki et al., 2017), alongside evidence that individuals with PTSD with versus without the dissociative subtype exhibit differential FC to other brain regions based on basolateral and centromedial divisions of the amygdala (Brown et al., 2014; Nicholson et al., 2015). In addition, in response to trauma‐related stimuli in individuals with PTSD, direction of amygdala FC to the PFC and brainstem depend on amygdala subregion distinctions (e.g., basolateral vs. centromedial nuclei (Rabellino et al., 2016)). In building upon these findings, the current study provides evidence that intra‐amygdala FC may be an important consideration for predicting variance in PTSD symptoms. In addition, atypical OFC responding (Huang et al., 2014; Thomaes et al., 2013) is documented in those with PTSD, with altered engagement of this region theorized to contribute to symptoms of anger, irritability, and recklessness often evident in the disorder (Weston, 2014). As highlighted earlier, atypical amygdala‐OFC FC is also documented in those with PTSD (Aghajani et al., 2016; Zhang, Wu, et al., 2016; Zhu et al., 2017). As the OFC is involved in appraisal of emotional states in conjunction with assessing reward and predictive value of stimuli, altered FC between the amygdala that detects emotional stimuli and the OFC that assigns value to this experience could result in atypical emotional responding (Phillips, Drevets, Rauch, & Lane, 2003). Similarly, decreased engagement of the DLPFC occurs in those with PTSD during exposure to negative images (Blair et al., 2013) and when trying to down‐regulate negative emotions (Rabinak et al., 2014). The DLPFC is involved in top‐down regulation of emotion and is involved in decision making and selection of strategies for emotion regulation (Lee & Seo, 2007; Yamagishi et al., 2016). Although not directly connected to the amygdala as much as ventral and medial portions of the PFC, the DLPFC modulates amygdala response in healthy individuals (Barbas, 2000; Ghashghaeia, Hilgetag, & Barbas, 2007; Stefanacci & Amaral, 2002). Therefore, atypical connectivity between the amygdala and the DLPFC as a predictor of PTSD severity substantiates the notion that PTSD is a disorder that is not just defined by aberrant bottom‐up generation of emotional states, but also disruption in the ability to regulate emotion through top‐down control (Fitzgerald, DiGangi, & Phan, 2018).

In addition, we found that amygdala FC with regions not typically explored in the etiology of PTSD helped in predicting PTSD severity. Specifically, amygdala FC with the caudate, cerebellum, superior parietal cortex, posterior cingulate cortex (PCC), and supramarginal gyrus was also within the top 10% of regions that contributed to correctly predicting PTSD symptoms. Limited studies have found disturbances in FC between the amygdala and these regions at rest. Nicholson and colleagues found that PTSD individuals with dissociation displayed enhanced amygdala FC with the superior parietal cortex (Nicholson et al., 2015). Enhanced connectivity with this region, which receives projections from visual and sensory cortices, may indicate disruptions in the ability to integrate sensory experiences with affective responses (Nicholson et al., 2015). With regard to the PCC, two investigations have found that greater PCC‐amygdala FC prospectively predicts greater symptoms six weeks (Lanius et al., 2010) and six months (Zhou et al., 2012) later as assessed by the Clinician‐Administered PTSD Scale (CAPS). The PCC is involved in the mentalizing process and plays a pivotal role in integrating information (Baliki, Mansour, Baria, & Apkarian, 2014). Thus, amygdala‐PCC connectivity as a predictor of PTSD variability may signal the disruption between detection of emotional salience (e.g., amygdala) and internal representation of this state (e.g., PCC) in those with PTSD. With regard to the role of the cerebellum in PTSD, its role in the disorder is still unclear despite a number of studies that have found altered amygdala‐cerebellum FC in those with PTSD (Brown et al., 2014; Stevens et al., 2013). Recently, altered cerebellum integrity was identified as a common feature of psychopathology (e.g., across internalizing, externalizing, and thought disorders) (Romer et al., 2018). As the cerebellum is involved in coordination and monitoring of incoming information (Romer et al., 2018), its role in PTSD pathophysiology may be linked to general deficits in the integration of affective experiences.

By contrast, to our knowledge no prior studies have documented atypical amygdala FC with the caudate and the supramarginal gyrus in those with PTSD. Nevertheless, altered connectivity between these regions and those closely linked to the amygdala has been found. First, Rabellino and colleagues found enhanced FC between the bed nucleus of the stria terminalis (BNST) and the caudate in those with PTSD (Rabellino et al., 2017). The BNST is a neural region closely connected to the amygdala that regulates the stress response (Choi et al., 2007). Enhanced FC between this region and the caudate, involved in action planning, associative learning, and inhibitory control (Provost, Hanganu, & Monchi, 2015), may signal atypical cognitive control over stress responses in those with PTSD. Second, decreased connectivity between the brainstem and supramarginal gyrus has also been found in those with PTSD (Harricharan et al., 2016). The amygdala receives direct connections from the brainstem in order to quickly process incoming sensory information that may signal threat. As the supramarginal gyrus integrates visual‐spatial information (Harricharan et al., 2016), decreased brainstem–supramarginal gyrus FC may portend atypical integration of sensory information.

Results of the present study should be considered in light of several limitations. First, although this sample, on average, did appear to suffer from clinically significant PTSD symptoms based on recommended PCL‐C cutoffs, there is substantial variability with regard to severity of illness. Thus, results should be interpreted with caution with regard to extending findings to chronically ill samples. Second, PTSD symptom severity was self‐reported. Future work should consider whether use of MVPA and amygdala FC validates prediction of clinician‐rated PTSD symptom severity. We also used a homogenous Caucasian adults’ sample for this study; thus, results may not extend to ethnic minorities. More work needs to be done to verify results using diverse populations. Finally, although we found evidence that whole‐brain amygdala FC predicted PTSD severity in a statistically significant manner, the strength of this correlation does not indicate that all the variance in PTSD severity can be explained by amygdala FC. Other individual factors need to be investigated as predictors of stress severity in trauma survivors, with one factor being the ways in which individual differences in FC with particular subnuclei of the amygdala—not investigated in this study—also correlates with PTSD severity, given differential roles of the amygdala divisions in fear learning (Díaz‐Mataix, Tallot, & Doyère, 2014). Future analyses should also consider other seed regions beyond the amygdala when investigating whole‐brain patterns of FC.

## 5 CONCLUSIONS

In conclusion, we demonstrated that MVPA in the context of amygdala FC is a valid approach for predicting severity of PTSD symptoms at the individual level. Although whole‐brain amygdala FC accurately predicted symptoms, amygdala FC within a fear acquisition, appraisal, and regulation network encompassing the amygdala, OFC, and DLPFC contributed to accurate prediction. In addition, regions not typically discussed in the etiology of PTSD, including the caudate, cerebellum, superior parietal cortex, PCC, and supramarginal gyrus, were among the top regions to contribute to the algorithm's success. In sum, results demonstrate that heterogeneous responses in amygdala FC that are spatially distributed are meaningful for the prediction of PTSD symptom severity, while also further supporting the specificity of fear acquisition and regulation neurocircuitries to predict individual differences in PTSD severity.

## CONFLICT OF INTEREST

The authors have no conflicts of interest or financial disclosures to report.

## AUTHOR CONTRIBUTIONS

JMF contributed to data analysis and writing of the manuscript. ELL, TAM, and WSP contributed to data collection and writing of the manuscript. CLL contributed to study design.

## Supporting information

Figure S1

## Data Availability

The data that support the findings of this study are available from the corresponding author upon reasonable request.
