# Supplementary figures and images for: Multi‐voxel pattern analysis of amygdala functional connectivity at rest predicts variability in posttraumatic stress severity
Source: Brain Behav. 2020 Jun 11;10(8):e01707. doi: 10.1002/brb3.1707 (PMC7428479; doi:10.1002/brb3.1707)

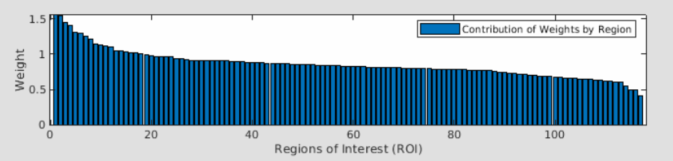

Supplement: Supplementary file 1 — Figure S1 [file BRB3-10-e01707-s001.tif]
